# Culture and real-time quantitative PCR to detect environmental nontuberculous mycobacteria in a clinical care center

**DOI:** 10.1016/j.temicr.2025.100016

**Published:** 2025-05-22

**Authors:** Meiyi Zhang, Brooke L. Smith, Rachel N. Wilsey, Tiana N.M. Koch, Elizabeth Dohanich, Pamela J. McShane, Jennifer R. Honda, Maria D. King

**Affiliations:** a Department of Biological and Agricultural Engineering, Texas A&M University, 333 Spence St, College Station, TX 77843, USA; b Department of Cellular and Molecular Biology, School of Medicine, University of Texas Health Science Center at Tyler, 11937 US Hwy 271, BMR Building, Tyler TX 75708, USA; c Department of Medicine, Section of Pulmonary and Critical Care, School of Medicine, University of Texas at Tyler, Tyler, TX, USA

**Keywords:** Nontuberculous mycobacteria, Aerosols, Environmental samples, Clinic, Ventilation

## Abstract

Nontuberculous mycobacteria (NTM) are opportunistic pathogens widely distributed in natural and human-built environments. Despite their environmental frequency, the airborne presence and distribution of NTM in clinical settings remain poorly understood. In this study, we assessed NTM in air and floor surface samples within a clinical care center using both culture-based and qPCR methods. Air and floor samples were collected across three days from patient rooms, a dictation room, and a hallway using a wetted wall cyclone (WWC), a gelatin filter-based air sampler, and foam swabs. Using the culture-based method, viable NTM were recovered from three floor surface swabs, while all other samples were culture-negative. qPCR analysis revealed frequent NTM detection in air samples, with a higher positivity rate from WWC samples compared to gelatin filters, despite total bacterial count (TBC) being two orders of magnitude higher in gelatin filter samples. The highest frequency of qPCR-tested NTM-positive samples was observed in the clinic hallway, suggesting that airflow dynamics and patient movement influence bacterial redistribution. Floor swabs showed transient NTM detection patterns, likely influenced by environmental deposition and routine cleaning. Despite confirmed *Mycobacterium avium* complex (MAC) infections among patients, *M. avium* was not detected in any environmental samples, suggesting species-specific aerosolization differences. Although viable NTM were not recovered from air samples, the high qPCR positivity suggests the potential presence of airborne NTM that may not have been captured by culture due to viability loss or sampling limitations. These findings emphasize the role of air sampling methodologies in capturing NTM and highlight the need to better understand the factors influencing the persistence and distribution of airborne NTM in clinical environments.

## 1. Introduction

Nontuberculous mycobacteria (NTM) are a diverse group of environmental bacteria within the genus *Mycobacteria* that have gained increasing recognition as respiratory significant pathogens (Honda et al., 2015). Unlike *Mycobacterium tuberculosis*, an obligate intracellular pathogen, NTM are widely distributed in both natural and human-built environments, including soil, dust, and water systems (Kaelin et al., 2020; Torvinen et al., 2010; Honda, 2023). While *M. tuberculosis* is primarily transmitted person-to-person through respiratory routes, NTM infection is mainly acquired through the inhalation of aerosolized NTM from environmental sources (Halstrom et al., 2015; Click, 2015; Jeon, 2019). A number of NTM species are capable of causing progressive pulmonary disease (PD), particularly in individuals with predisposing conditions such as chronic obstructive pulmonary disease (COPD), cystic fibrosis (CF), and bronchiectasis (Griffith et al., 2007). Although not as common, NTM can also cause skin and soft tissue infections, lymphadenitis, and disseminated infections in immunocompromised patients (Gonzalez-Santiago and Drage, 2015). In the United States (U.S.), the prevalence of NTM PD has increased over the last decades, with an estimated 86,000 cases in 2010 and a projected increase of 8.2 % per year (Adjemian et al., 2012; Strollo et al., 2015). The economic burden associated with NTM infections is substantial, with estimated annual healthcare costs exceeding $815 million, primarily due to prolonged treatment regimens and hospitalizations (Strollo et al., 2015). Given the increasing prevalence of NTM PD and its significant public health impact, there is a continued need for improved surveillance and detection strategies to mitigate NTM-associated risks, particularly in healthcare settings.

Clinical care centers, including hospitals, outpatient clinics, and long-term care facilities, represent critical environments where patients may be exposed to NTM, particularly through airborne transmission (Desai and Hurtado, 2018). Recent studies demonstrate aerosolization of NTM from colonized sources, including showerheads, hot tubs, therapy pools, heater-cooler devices, and humidifiers (Kaelin et al., 2020; Shen et al., 2022; L.M. Feazel et al., 2009; Glazer et al., 2007; Hamilton and Falkinham, 2018; Thomson et al., 2013). A few studies have reported the presence of viable NTM species in air samples collected from various indoor environments such as residential homes and hospitals, suggesting that airborne transmission may play a more significant role in NTM exposure than previously recognized (Kaelin et al., 2020; Thomson et al., 2013). The ability of NTM to resist desiccation and remain suspended in air is attributed to their thick, hydrophobic, lipid-rich cell wall that enhances environmental persistence (Jarlier and Nikaido, 1994; Pereira et al., 2020). Furthermore, their propensity to form biofilms on different surfaces provides a continuous source for NTM to become aerosolized, further complicating disinfection efforts (Faria et al., 2015). Standard disinfection protocols, including chlorine-based treatments, are often ineffective against NTM due to their intrinsic resistance mechanisms (Ditommaso et al., 2020; Taylor et al., 2000). As a result, airborne NTM, particularly in clinical care centers where immunocompromised individuals may be at greater risk, represents an unstudied yet potentially critical exposure route that requires further investigation.

The objective of this study was to detect and quantify NTM in environmental air and surface samples collected from a clinical care center using both culture-based method and qPCR by sampling patient rooms, a dictation room, and a hallway during a three-day period. The findings of this study will enhance understanding of NTM distribution in healthcare environments, informing infection control strategies and environmental surveillance efforts. Given the increasing clinical significance of NTM infections and the challenges associated with their environmental persistence, this study provides valuable insights to support improved public health interventions and infection prevention measures.

## 2. Materials and methods

### 2.1. Sample collection

**Fig. 1.**
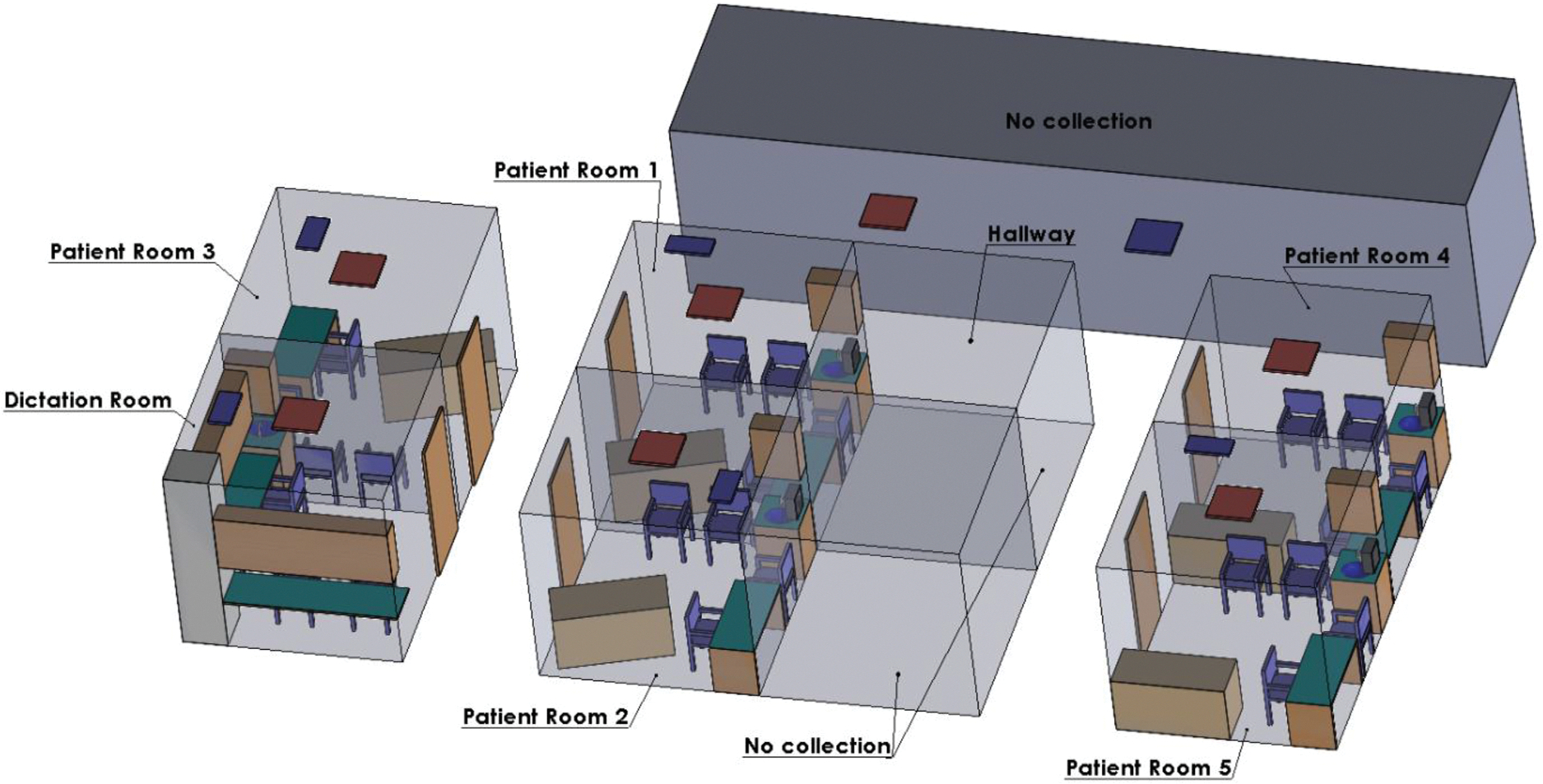
Isometric view of the clinic where air and surface collection took place.

Aerosol and surface samples were collected from a pulmonary clinic within a clinical care center located in Texas, U.S. in January 2024. Environmental sampling was conducted in multiple locations, including patient rooms (PR 1, 2, 3, 4, 5), a dictation room, and a hallway. The frequency of sampling varied based on room availability and patient placement, which was determined by physicians. A geometric model of the sampling site was created in SolidWorks to illustrate room locations and furniture arrangements (Fig. 1). Each patient room had a standardized layout consisting of an examination bed, a desk and chair for physicians, a sink with a top cabinet, three visitor chairs, an air inlet/diffuser, and an exhaust system (except for PR 4 which lacked an exhaust). The dictation room contained desks, chairs, top cabinets, an air inlet/diffuser, and an exhaust, while the hallway included an air inlet/diffuser and an exhaust. Each room measured 8.4 m^2^ in area and 22.7 m^3^ in volume. Doors remained closed during patient visits and sampling. Patient rooms and hallways were cleaned and disinfected once every evening, Monday through Friday. High-touch surfaces in patient rooms are disinfected using a hydrogen peroxide-based disinfectant, while floors are cleaned with a maintenance floor cleaner containing nonionic surfactants and alcohol.

Air samples were collected using an MD8 Airport Portable Air Sampler (Sartorius, Goettingen, Germany) operating at 50 L/min for 20 min onto gelatin membrane filters (Sartorius, Goettingen, Germany) and a wetted wall cyclone (WWC) developed by McFarland et al. (McFarland et al., 2010) running at 100 L/min into autoclaved Milli-Q water. Prior to air collection, the WWC units were decontaminated by sequentially flushing the system with 10 % bleach, isopropanol, and sterile Milli-Q water for 10 min. Following collection, gelatine membrane filters were immediately dissolved in 10 mL phosphate-buffered saline (PBS) buffer and stored at −20 °C. Surface samples were collected prior to air sampling, from a 1 ft^2^ (0.093 m^2^) floor area directly below the air diffuser using a Foamtip swab (Chemtronics, Kennesaw, GA). Swabs were stored in 2 mL PBS buffer, kept on ice, and vortexed thoroughly before processing. Sampling was conducted over three days, with each collection event comprising two air samples (one gelatin filter and one WWC) and one surface swab per location and time point (Day 1, Day 2 AM, Day 2 PM, and Day 3). A total of 19 gelatine filter air samples, 19 WWC air samples, and 19 surface swab samples were collected. For patient room sampling on Day 1 and 2, air and surface samples were obtained immediately after patient visits. No patients were present on Day 3.

After each patient visit, air sampling was conducted in the respective patient room. No samples were taken directly from the patients. Both air and surface samplings were conducted after they left. Therefore, the study was exempted from IRB. To assess potential correlation between NTM detection and patient characteristics, anonymized patient data was recorded, including gender, age, and the presence or absence of clinically confirmed *Mycobacterium avium* complex (MAC) pulmonary disease. Notably, all patients involved in this sampling event were diagnosed with ongoing bronchiectasis, a known comorbidity of NTM infected individuals (Yin et al., 2021).

### 2.2. DNA extraction and bacteria quantification by qPCR

DNA extraction was performed on the liquid resuspensions obtained from air and surface samples using a previously established alkaline lysis method (Zhou et al., 1990). Following collection, gelatin membrane filters were dissolved in PBS buffer, and swabs were stored in PBS buffer and vortexed thoroughly. These suspensions were initially centrifuged, and the resulting pellets were resuspended in 300 μL of Tris-EDTA-NaCl buffer. To enhance nucleic acid recovery, 150 μL of 3 N sodium acetate was added, followed by an additional centrifugation step. The supernatants were carefully transferred to fresh Eppendorf tubes, and 10 μL of Poly Acryl Carrier (PAC, Molecular Research Center) introduced to facilitate nucleic acid concentration. Precipitation was performed by adding 1 mL of 100 % isopropanol, and after centrifugation, the supernatants were discarded. Pellets were resuspended in 1 mL of cold 100 % ethanol, followed by another centrifugation step. The ethanol was subsequently removed, and the pellets were air-dried before being reconstituted in 50 μL of sterile Milli-Q water and stored at −20 °C until use.

The sequences of primers for detecting total bacterial count (TBC), *Mycobacterium* sp., and *M. avium* are shown in Table 1, and the abundances of the target of interest were quantified according to previous studies (King and McFarland, 2012; Baig et al., 2022). *M. avium* was selected as a representative species of MAC due to its high prevalence within the complex and its frequent detection in clinical and environmental samples. Experimental conditions of the qPCR method were reported in accordance with the Minimum Information for Publication of Quantitative Real-Time PCR Experiments (MIQE) guidelines (Bustin et al., 2009). For gene amplification, the reaction mixture (10 μL total volume) consisted of 5 μL of 2 × SYBR Green PCR Master Mix (Applied Biosystems, Foster City, CA), 1 μL each of 100 μM forward and reverse primers (primer sequences listed in Table 1), and 3 μL of extracted DNA. Real-time qPCR was carried out using the QuantStudio 3 Real-Time PCR System (Applied Biosystems, Waltham, MA), with a melting curve analysis performed over a temperature range of 60 to 90 °C. For TBC, the thermocycling protocol consisted of an initial denaturation at 95 °C for 10 min, followed by 40 cycles of 95 °C for 15 s and 60 °C for 60 s. For *Mycobacterium* sp., cycling conditions included a 15-min hot start at 94 °C, followed by 40 cycles of 94 °C for 30 s, 63 °C for 15 s, and 72 °C for 40 s. For MAC-specific assay, the protocol consisted of an initial denaturation of 94 °C for 10 min, followed by 40 cycles of 94 °C for 15 s and 60 °C for 45 s. Samples were considered positive if amplification was detected at a cycle threshold (Ct) value of less than 40 (Ct < 40). For positive samples, Ct values were used to determine the concentration of target organisms, expressed as Gene Copy Number (GCN) per mL, based on standard curves as shown in Supplementary Material Figure S1. The Limit of Detection (LOD) were 13 GCN/mL for TBC and 31 GCN/mL for NTM. The final GCN per cubic meter of air or square meter of floor surface was calculated by accounting for the sample volume and the total volume of air collected. The WWC collection details were included in the Supplementary Material Table S1.

### 2.3. Culture and NTM species identification by sequencing

The 19 gelatine filter air samples, 19 WWC air samples, and 19 surface swab samples were cultured for viable NTM using previously published methods (Virdi et al., 2021; Honda et al., 2016). Briefly, samples were disinfected with 1 % cetylpyridinium chloride (CPC), a treatment that minimizes the growth of non-mycobacterial organisms with minimal impact on NTM viability, and cultured onto Middlebrook 7H10 agar plates containing oleic acid/glycerol enrichment with and without amphotericin B (Radomski et al., 2010). After incubation at either 30 °C or 37 °C for 21 days, single colony picks representing each morphology consistent with NTM (total of 4 isolates) were grown in Middlebrook 7H9 broth until turbid. Of the turbid culture 1 mL was centrifuged at 1300 × *g* for 2 min and the resulting pellet was used for DNA extraction following a specific protocol for mycobacterial cell wall lysis (Epperson and Strong, 2020). NTM identification at the species level was conducted by sequencing a 711 bp *rpo*B gene region using Sanger sequencing (Quintara Biosciences, CA) (Adékambi et al., 2003; Walters et al., 2016). The results from sequencing were controlled for quality via trimming and compared to *rpo*B reference sequences using the BLAST algorithm provided by NCBI GenBank.

### 2.4. Statistical analyses

Statistical analyses were conducted to assess the effect of patient age and MAC diagnosis on NTM concentrations in air samples and the likelihood of NTM detection. Given the non-normal distribution of NTM concentrations, Mann-Whitney U tests were used to compare air sample NTM levels between patient age groups (≤75 years vs. >75 years) and patients with and without a clinical MAC diagnosis. To evaluate the association between these factors and the presence or absence of NTM in air samples, Fisher’s exact test was applied due to the small sample size and low expected frequencies in contingency tables. Specifically, Fisher’s exact tests were used to determine whether patient age and MAC diagnosis were significantly associated with NTM detection (positive vs. negative samples). All statistical tests used a significance threshold of *p* < 0.05. Analyses were performed using GraphPad Prism (GraphPad Software, San Diego, CA).

## 3. Results

Sampling was conducted over three days in January 2024 (winter season): Day 1, Day 2 (morning AM and afternoon PM sessions), and Day 3. On Days 1 and 2, the clinic was fully operational with approximately 15 to 20 patients present each day, whereas on Day 3, only physicians were present. Air and floor surface samples were collected immediately after a patient exited the room and relevant patient information, including gender, age, and clinically confirmed MAC status, was recorded anonymously. A total of 19 sampling events were conducted, each involving air sampling using a gelatin filter-based air collector and a WWC, as well as a floor surface swab (Table 2). Among these, 10 sampling events involved patients, consisting of 8 females and 2 males, aged 60 to 81 years, with MAC infection status classified as positive, negative, or unknown.

Among the 57 total samples tested, culturable NTM were recovered from only three floor surface swabs and none of the air samples (Table 2). The NTM culture-positive floor samples were collected from the dictation room and PR 3 on Day 1 and the hallway on Day 2 PM. Species identification revealed the presence of *Mycobacterium gallinarum* in the dictation room, *Mycobacterium iranicum* in PR 3, and *Mycobacterium phocaicum* and *Mycobacterium porcinum* in the hallway. In contrast, qPCR targeting the *Mycobacterium* 16S rRNA gene detected *Mycobacterium* sp. in 26 (45.6 %) out of 57 samples, including 5 (26.3 %) from gelatin filter-based air samples, 14 (73.7 %) from WWC air samples, and 7 (36.8 %) from floor surface swabs (Table 2). To confirm that these detections represented NTM and not *M. tuberculosis*, an additional qPCR assay specific for *M. tuberculosis* was performed, yielding negative results for all samples. Further qPCR analysis using a MAC-specific assay also returned negative results, even in samples collected from rooms where individuals with clinically confirmed MAC infections had been present.

**Fig. 2.**
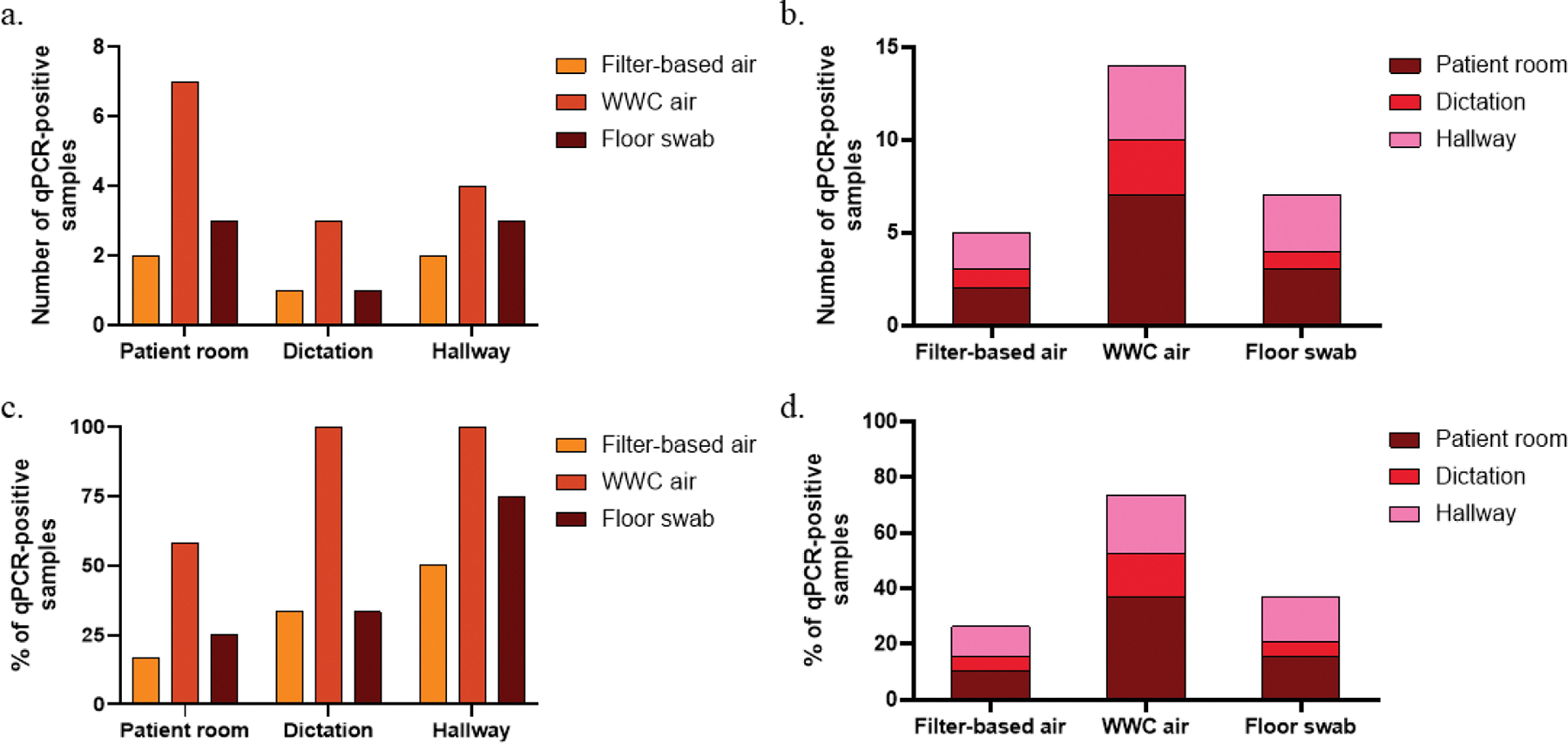
Frequency and percentage of NTM-positive samples detected using qPCR based on types of locations (patient room, dictation, and hallway) or sampling methods (air collected with filter-based method, air collected with WWC, and swab of floor surface). Frequency of positive samples (a) based on sampling methods, grouped by locations, and (b) vice versa. Percentage of positive samples (c) based on sampling methods, grouped by locations, and (d) vice versa.

Among the 19 total collection events, 12 were conducted in PR, three in the dictation room, and four in the hallway. The WWC air sampler detected the highest number of qPCR NTM-positive samples in PR (Fig. 2a), suggesting its superior ability to capture aerosolized NTM. When stratified by sampling technique, WWC air samples yielded the highest qPCR NTM positivity rate, while filter-based air samples detected the least (Fig. 2b). However, after normalizing to the total number of collection events per location, the proportion of positive samples in PR was 53 % (Fig. 2c). When comparing NTM qPCR detection frequency across locations, the hallway exhibited the highest percentage of positive samples, followed by the dictation room and then PR, regardless of the sampling method. Similarly, as shown in Fig. 2d, after normalization the qPCR NTM positivity rate was the highest from WWC air samples and lowest from filter-based air samples. These findings suggest that WWC air sampling may provide improved detection sensitivity for airborne NTM compared to the gelatin filter-based method, while floor surface swabs remain useful for detecting settled NTM.

**Fig. 3.**
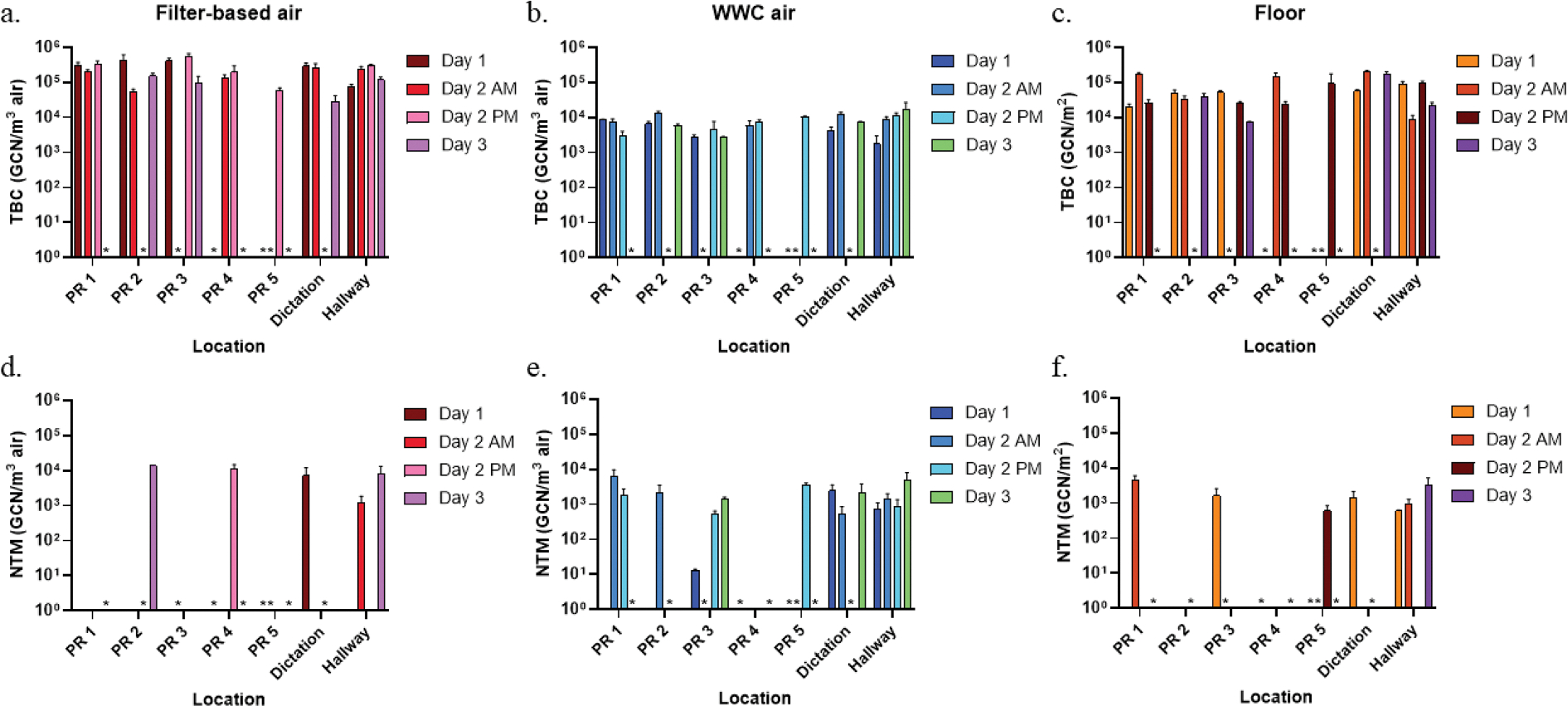
Quantification of Total Bacteria Concentration (TBC) by qPCR during the three-day collection period in (a) air collected with filter-based method, (b) air collected with WWC, and (c) swab of floor surface. Quantification of NTM by qPCR during the three-day collection period in (d) air collected with filter-based method, (e) air collected with WWC, and (f) swab of floor surface. PR represents patient room.

To further assess bacterial concentration differences among collection events, quantification of TBC and NTM was performed using qPCR (Fig. 3). TBC was successfully quantified in all samples, whereas NTM was detected in 46 % of samples (26/57). TBC levels remained relatively stable across the three-day sampling period and across locations, fluctuating within two orders of magnitude between 10^4^ and 10^5^ GCN/ m^3^ for air samples collected using gelatin filters (Fig. 3a). A similar trend was observed in WWC-collected air samples, where TBC ranged between 1.77 × 10^3^ and 1.75 × 10^4^ GCN/m^3^ (Fig. 3b). In contrast, floor swabs exhibited greater variability in TBC concentrations, ranging from a low of 7.24 × 10^3^ GCN/m^2^ in PR 3 on Day 3 to a high of 2.05 × 10^5^ GCN/m^2^ in the dictation room on Day 2 AM (Fig. 3c). At certain locations, such as PR 1, PR 4, and the hallway, TBC levels fluctuated both throughout the three-day period and within the same day (Day 2), suggesting dynamic bacterial deposition patterns. For NTM quantification, all five positive filter-collected air samples exhibited NTM concentrations of around 10^4^ GCN/m^3^, except for the hallway on Day 2 AM, where levels were slightly lower at 1.17 × 10^3^ GCN/m^3^ (Fig. 3d), although the difference is not statistically significant (*p* = 0.059). In WWC-collected air samples, NTM concentrations were lower than those observed with gelatin filter collection (highest level with WWC air at 6.24 × 10^3^ GCN/m^3^ versus with filter-collected air at 1.39 × 10^4^ GCN/m^3^), yet WWC demonstrated a higher frequency of NTM detection (Fig. 3e). The lowest NTM concentration in WWC-collected air samples was found at PR 3 on Day 1 at around two magnitudes lower than the other days. Among floor swabs, the highest NTM concentration was detected in PR 1 on Day 2 AM at 4.57 × 10^3^ GCN/m^2^, while the lowest was found in the hallway on Day 1 at 5.98 × 10^2^ GCN/m^2^ (Fig. 3f). Notably, NTM concentrations did not significantly differ between patient rooms and non-patient areas (dictation room and hallway), nor between operational and non-operational clinic periods with the exception of PR 2 in which NTM was detected in air samples collected via gelatin filters only on Day 3, a non-operational clinic period.

Statistical analyses were performed to assess potential associations between NTM levels in air samples and patient characteristics, specifically patient age groups (≤75 years vs. >75 years) and with or without a clinical MAC diagnosis (Fig. S2). Neither factor was found to significantly influence NTM concentrations (MAC diagnosis: *p* > 0.99; age: *p* = 0.41). Similarly, Fisher’s exact tests were conducted to evaluate whether NTM detection (positive vs. negative samples) was significantly associated with these patient factors, but no statistically significant relationships were observed (MAC diagnosis: *p* = 0.44; age: *p* > 0.99). It is important to note that the sample size for these analyses was limited (*n* = 10 for age association; *n* = 9 for MAC diagnosis status), which may reduce statistical power and the ability to detect subtle differences. While these findings do not suggest strong associations within the current dataset, larger studies may be necessary to further evaluate these potential relationships.

## 4. Discussion

This study evaluated the presence and distribution of NTM in air and floor surface samples within a pulmonary clinic using both microbiological culture for viable NTM and qPCR methods. Despite the recognized persistence of NTM in healthcare environments, a gap remains in understanding the prevalence and distribution of NTM throughout clinical care centers, particularly in air and on high-touch surfaces. Culture-based techniques have traditionally been the gold standard for NTM identification, allowing for species-level differentiation and antimicrobial susceptibility testing (Radomski et al., 2010; Pennington et al., 2021; Brown-Elliott et al., 2012; Kostecki et al., 2024). However, these methods are inherently slow, requiring several weeks for NTM growth, and may underrepresent total NTM presence as a result of selective recovery of certain strains (Mercaldo et al., 2023; Zhang et al., 2024). Real-time qPCR has been extensively used as an alternative, culture-independent, rapid, and sensitive method for NTM detection in environmental samples (Dziedzinska et al., 2016). This molecular approach is particularly advantageous for identifying slow-growing or non-culturable NTM strains (Torvinen et al., 2010). However, qPCR does not distinguish between viable and non-viable bacteria, which may limit its utility in assessing infection risk. Given the complementary strengths and limitations of these methodologies, employing both culture and qPCR methods for environmental NTM detection may provide a more comprehensive assessment of their prevalence in clinical care centers. Our findings demonstrated that NTM DNA was detectable in nearly half of all environmental samples collected, with the highest detection frequency observed in airborne samples collected using the WWC sampler. NTM positivity varied substantially between the two detection methods, with qPCR detecting NTM in 45.6 % of samples (26/57) and culture-based methods detecting NTM in only 5.3 % of samples (3/57). This may be due to qPCR detecting non-viable NTM or detecting NTM that was not culturable.

The clinic hallway exhibited the highest frequency of NTM-positive samples compared to PR and the dictation room. While our results did not show a statistically significant correlation between patient presence and NTM detection, the elevated detection in hallway samples may reflect the influence of airflow dynamics or particle resuspension. Hallways may function as transitional zones where bioaerosols accumulate due to air currents or physical disturbance, though further investigation is needed to confirm these mechanisms. The continuous detection of NTM in both air and surface samples within hallways during the three days further supports this hypothesis. Additionally, WWC-based air sampling yielded a higher frequency of NTM-positive samples compared to gelatin filter-based air sampling, suggesting that WWC may provide a more efficient method for NTM detection. Despite the higher NTM detection frequency with WWC, TBC concentrations were overall two orders of magnitude higher in gelatin filter samples. This discrepancy likely arises from the differences in collection mechanisms and bacterial properties. Gelatin filters efficiently capture a broad range of airborne bacteria, including both hydrophilic and hydrophobic species, while WWC, utilizing high-velocity air and liquid impaction, may preferentially capture hydrophobic bacteria such as NTM, which are more likely to remain airborne and evade gelatin filter collection (McFarland et al., 2010; Falkinham, 2021). Similar observations were reported by Wu *et al*., who found higher bacteria and nucleotide concentrations in air samples collected with gelatin filters compared to liquid impingers (Wu et al., 2010). While WWC samples exhibited a higher frequency of NTM detection, NTM concentrations in NTM positive samples were comparable across both air sampling methods. These findings underscore the importance of sampling method selection, as different techniques may preferentially capture bacterial subgroups based on their physical and biological characteristics (Dybwad et al., 2014; Abeykoon et al., 2022). Interestingly, NTM quantification results from hallway floor swabs revealed a transient pattern, with a positive detection on Day 2 AM but a negative result on Day 2 PM, followed by a higher concentration on Day 3. This fluctuation may be attributed to the swabbing process itself removing most of the deposited NTM, with the possibility that airborne NTM requires additional time to resettle onto surfaces. These results suggest that the process of NTM accumulation on floors is dynamic and may be influenced by the timing of sampling relative to environmental factors such as airflow patterns, human activity, and particle resuspension.

While extensive research has been conducted on NTM presence in water systems (Kaelin et al., 2020; Honda, 2023; Glazer et al., 2007; Dutil et al., 2007; Kostecki et al., 2024; Ouradou et al., 2023), studies focusing on airborne NTM within clinical environments remain limited. One of the few investigations addressing NTM transmission in healthcare settings was conducted by Gross et al., who examined potential healthcare-associated NTM transmission in a cystic fibrosis (CF) clinic using whole-genome sequencing and epidemiologic analysis (Gross et al., 2023). Although their study could not confirm healthcare-associated NTM transmission, they identified genetically similar *M. intracellulare* ssp. *chimaera* respiratory isolates and hospital water biofilm isolate, suggesting possible environmental acquisition of the strain. This finding raises important questions about the role of bioaerosols in NTM dissemination within clinical settings, as environmental sources such as air and surfaces were not implicated in their study. Our study addresses this gap by providing quantitative data on NTM detection in air and surface samples from a clinical environment, highlighting the need to further investigate the role of aerosolization in NTM transmission in the healthcare settings. Given that we did not detect *M. avium* in any samples, even with the presence of patients with clinically confirmed MAC infections, it is possible that certain NTM species exhibit selective aerosolization, where differences in cell wall properties such as hydrophobicity and waxy quality may influence their ability to partition into aerosols - a phenomenon previously proposed in several studies (L.M. Feazel et al., 2009; Angenent et al., 2005). Factors such as hydrophobicity, cell wall composition, and biofilm formation likely contribute to differences in airborne dispersal among NTM species (Falkinham, 2003). Another notable observation was the low NTM detection rate from floor surface swabs, which may be attributed to the clinic’s daily floor cleaning regimen using a disinfectant containing nonionic surfactants and alcohol. These agents are known to disrupt bacterial membranes, reduce microbial viability, and alter surface adhesion properties (Moore et al., 2006; Glover et al., 1999; Brown and Jaffé, 2006), potentially reducing NTM recovery from floors compared to air samples. Additionally, residual cleaning agents may have further inhibited NTM persistence, reinforcing the importance of environmental hygiene in limiting bacterial reservoirs in healthcare settings. While this cleaning is expected to reduce microbial load broadly, the TBC on floors was not consistently lower. This discrepancy may reflect differences in the susceptibility or recoverability of various bacteria following cleaning. For example, some surface-associated bacteria may persist or be more easily recovered, while NTM may exist at lower abundance or be more affected by physical or chemical disruption during cleaning. Finally, our study considered the potential influence of the HVAC system on bioaerosol distribution and bacterial resuspension. Measurements of air velocity at each collection site (Table S2) showed that most locations had one air diffuser and one exhaust vent, except for PR 4, which lacked an exhaust vent. The absence of an exhaust in PR 4 may have affected air circulation and particle removal, potentially influencing local NTM concentrations. The role of ventilation systems in modulating bioaerosol dynamics has been well-documented in previous studies (Baig et al., 2022; Kumar and King, 2022; Luongo et al., 2016), and our findings highlight the need for further investigation into HVAC-mediated bacterial transport in clinical settings.

This study is the first, to our knowledge, to investigate the presence of airborne environmental NTM collected after patient visits in a clinic environment. However, several limitations should be noted. First, air sampling was conducted after patients exited the room due to the high noise level of the equipment. While this approach minimized patient discomfort, it may not fully capture the potential contribution of patients actively exhaling NTM into the air. Second, the small number of patient subjects limits the statistical power of our analysis, restricting our ability to evaluate associations between patient characteristics and NTM detection in environmental samples. Third, the study was conducted in a single healthcare center over a limited timeframe, which may constrain the generalizability of our findings. Sampling was conducted over three days in January in Texas; although indoor temperature and humidity were controlled, unmeasured outdoor environmental factors may have influenced NTM presence. Prior studies suggest that environmental variables such as temperature and precipitation can impact NTM distribution (Blanc et al., 2021; Campbell et al., 2024). While seasonal variation was not assessed in this preliminary study, future longitudinal sampling across different seasons is planned to better characterize the potential influence of environmental conditions on airborne and surface-associated NTM presence. Additionally, including a larger patient cohort and incorporating real-time air sampling during patient presence will be important to better understand the role of human-associated aerosolization in NTM dissemination.

## 5. Conclusions

This study provides new insights into the NTM presence and distribution in a clinical care center. NTM was more frequently detected in air samples, particularly when using the WWC method. While culture-based methods yielded limited viable NTM recovery, qPCR analysis revealed a higher frequency of NTM detection, highlighting the value of molecular techniques for environmental surveillance. The hallway showed the highest frequency of NTM-positive samples, suggesting a role for airflow dynamics and human activities in bacterial redistribution. Despite the presence of clinically confirmed MAC-infected patients, respiratory important MAC species were not detected, suggesting possible selective aerosolization of NTM species. TBC remained relatively stable across samples, while NTM detection varied by sample type and location. These findings highlight the dynamic nature of NTM distribution in healthcare environments and the need for further studies incorporating real-time bioaerosol monitoring, seayysonal variation, expanded patient cohorts, and HVAC assessments to better understand exposure risks and inform infection control strategies.

## Supplementary Material

Supplementary Materials

Supplementary materials

Supplementary material associated with this article can be found, in the online version, at doi:10.1016/j.temicr.2025.100016.

## Figures and Tables

**Table 1:** Primer sequences for TBC, *Mycobacterium* sp., and *M. avium* amplifications.

Primer Name	Sequence	Target organisms	Length (bp)	Reference

16S 1048	GTGSTGCAYGGYTGTCGTCA	Total bacteria	146	(King and McFarland, 2012)
16S 1194	ACGTCRTCCMCACCTTCCTC			
pMyc14	GRGRTACTCGAGTGGCGAAC	*Mycobacterium* sp.	209	(Dutil et al., 2007)
pMyc7	GGCCGGCTACCCGTCGTC			
8F	AGAGTTTGATCCTGGCTCAG	*M. avium*	192	(L.M. Feazel et al., 2009)
MAV199R	ACCAGAAGACATGCGTCTTG			

**Table 2:** Detection of NTM in air and floor samples from each collection event with sampling information based on the qPCR and culture results.

Time	Location	Patient information (if patient present)			Detection of NTM in samples					
					qPCR			Culture		
					Air		Floor Swab	Air		Floor Swab
			
		Gender	Age (yr)	Clinically tested MAC status	Filter-based	WWC		Filter-based WWC		

Day 1	PR1	Female	81	Unknown	ND	ND	ND	ND	ND	ND
Day 1	PR2	Female	78	Positive	ND	ND	ND	ND	ND	ND
Day 1	Dictation				Positive	Positive	Positive	ND	ND	Positive
Day 1	PR3	Female	79	Negative	ND	Positive	Positive	ND	ND	Positive
Day 1	Hallway				ND	Positive	Positive	ND	ND	ND
Day 2 AM	PR1	Female	63	Negative	ND	Positive	Positive	ND	ND	ND
Day 2 AM	PR2	Female	77	Positive	ND	Positive	ND	ND	ND	ND
Day 2 AM	Dictation				ND	Positive	ND	ND	ND	ND
Day 2 AM	PR4	Female	65	Positive	ND	ND	ND	ND	ND	ND
Day 2 AM	Hallway				Positive	Positive	Positive	ND	ND	ND
Day 2 PM	PR1	Female	61	Negative	ND	Positive	ND	ND	ND	ND
Day 2 PM	PR5	Female	80	Intermittently positive	ND	Positive	Positive	ND	ND	ND
Day 2 PM	PR3	Male	60	Negative	ND	Positive	ND	ND	ND	ND
Day 2 PM	PR4	Male	71	Positive	Positive	ND	ND	ND	ND	ND
Day 2 PM	Hallway				ND	Positive	ND	ND	ND	Positive
Day 3	PR2				Positive	ND	ND	ND	ND	ND
Day 3	Dictation				ND	Positive	ND	ND	ND	ND
Day 3	PR3				ND	Positive	ND	ND	ND	ND
Day 3	Hallway				Positive	Positive	Positive	ND	ND	ND

* PR: Patient Room.

* ND: Not Detected (under detection limit).

## Data Availability

Data will be made available on request.
