# Supplementary material for: Culture and real-time quantitative PCR to detect environmental nontuberculous mycobacteria in a clinical care center
Source: Total Environ Microbiol. Author manuscript; Available in PMC 2026 Feb 19. (PMC12916027; doi:10.1016/j.temicr.2025.100016)
Supplement: Supplementary Materials [file NIHMS2139846-supplement-Supplementary_Materials.docx]

**Supplementary Materials**

**Table S1**. Air volume and liquid output collected by WWC for the nineteen WWC air samples.

| Time | Location | WWC collection time (min) | WWC air collected (m^3^) | WWC liquid output (mL) |
| --- | --- | --- | --- | --- |
| Day 1 | PR1 | 20 | 2 | 7.9 |
| Day 1 | PR2 | 20 | 2 | 8.2 |
| Day 1 | Dictation | 15 | 1.5 | 5.5 |
| Day 1 | PR3 | 30 | 3 | 1.7 |
| Day 1 | Hallway | 20 | 2 | 7.7 |
| Day 2 AM | PR1 | 20 | 2 | 9.3 |
| Day 2 AM | PR2 | 20 | 2 | 7.4 |
| Day 2 AM | Dictation | 20 | 2 | 7.8 |
| Day 2 AM | PR4 | 20 | 2 | 9.5 |
| Day 2 AM | Hallway | 20 | 2 | 8.3 |
| Day 2 PM | PR1 | 20 | 2 | 7.4 |
| Day 2 PM | PR5 | 20 | 2 | 7.4 |
| Day 2 PM | PR3 | 20 | 2 | 7.9 |
| Day 2 PM | PR4 | 20 | 2 | 7.8 |
| Day 2 PM | Hallway | 20 | 2 | 7.9 |
| Day 3 | PR2 | 20 | 2 | 6.6 |
| Day 3 | Dictation | 20 | 2 | 6.4 |
| Day 3 | PR3 | 20 | 2 | 6.9 |
| Day 3 | Hallway | 20 | 2 | 6.5 |
| * PR: Patient Room. | | | | |


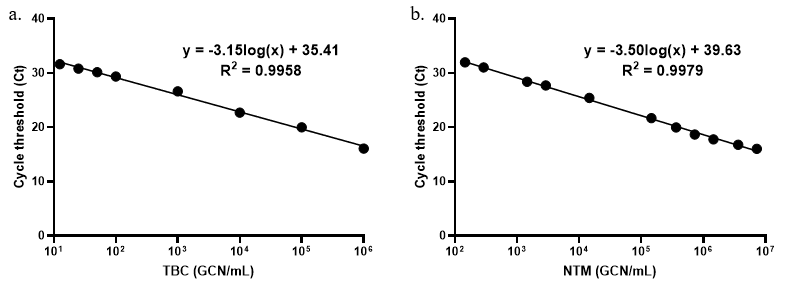


**Figure S1.** qPCR standard curves for (a) TBC and (b) NTM, with corresponding regression equation and R^2^ values. The amplification efficiencies were 107.5% for TBC and 93.1% for NTM.


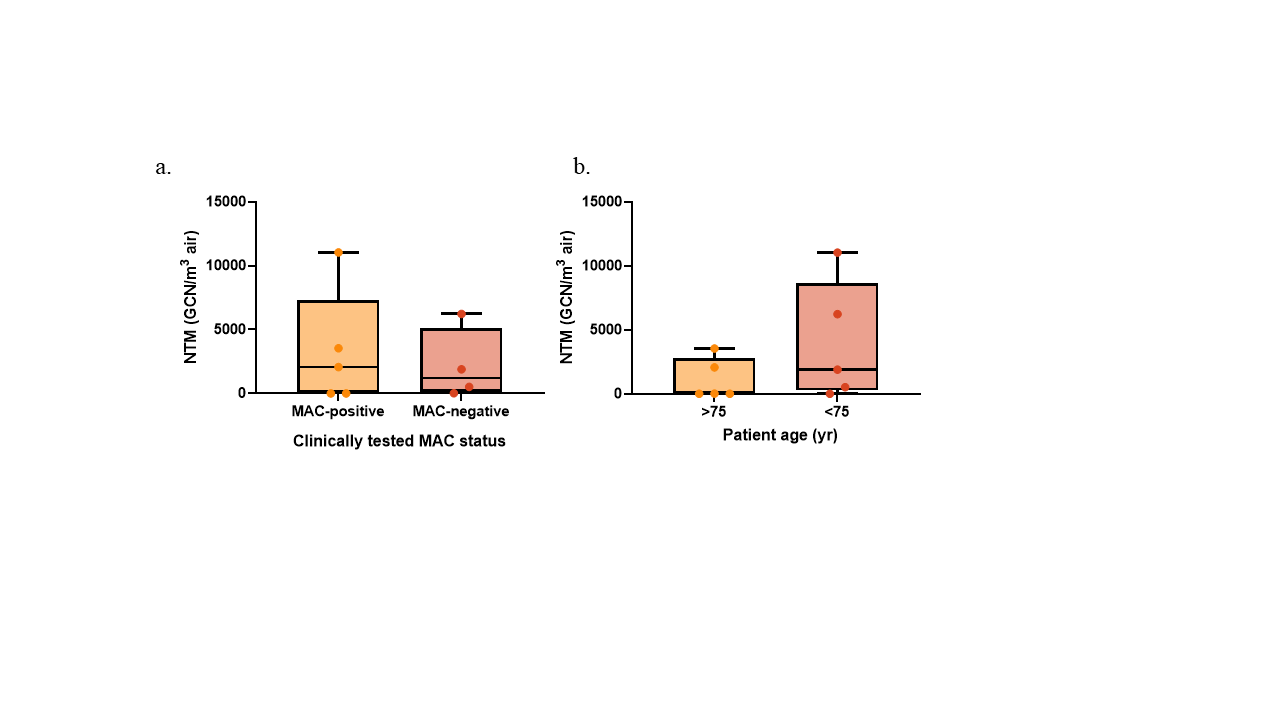


**Figure S2.** NTM concentrations in air samples that were collected when patients were previously present at the sampling location, grouped by (a) if the patient was clinically confirmed MAC-positive or -negative and (b) patients’ age above or below 75 years old.

**Table S2**. Air velocity measurement from the ventilation system consisting of the diffuser and exhaust (if present) at three pre-selected points (left, center, and right) at each sampling site.

|  | Air velocity (m/s) | | | | | |
| --- | --- | --- | --- | --- | --- | --- |
|  | Diffuser | | | Exhaust | | |
| Location | Left | Center | Right | Left | Center | Right |
| Hallway | 0.10 | 0.05 | 0.06 | 0.08 | 0.15 | 0.11 |
| Dictation | 0.14 | 0.03 | 0.07 | 0.03 | 0.01 | 0.01 |
| PR3 | 0.04 | 0.03 | 0.02 | 0.04 | 0.05 | 0.14 |
| PR2 | 0.04 | 0.04 | 0.01 | 0.01 | 0.01 | 0.01 |
| PR4 | 0.03 | 0.04 | 0.04 | Not present | | |
| PR1 | 0.03 | 0.01 | 0.04 | 0.02 | 0.01 | 0.01 |
| * PR: Patient Room. | | | | | | |
